# Supplementary material for: Single Cell Transcriptomic and Chromatin Profiles Suggest Layer Vb Is the Only Layer With Shared Excitatory Cell Types in the Medial and Lateral Entorhinal Cortex
Source: Front Neural Circuits. 2022 Jan 26;15:806154. doi: 10.3389/fncir.2021.806154 (PMC8826650; doi:10.3389/fncir.2021.806154)
Supplement: Supplementary file 14 [file Data_Sheet_1.ZIP › MEC_Sup_vs_LEC_Sup_Motifs/homerResults/motif1.similar.html]

motif1

## Information for motif1

G
C
T
A
T
A
C
G
A
G
T
C
C
G
T
A
A
C
T
G
A
C
T
G
A
C
G
T
A
C
T
G
A
G
C
T
C
T
G
A
  
Reverse Opposite:  

G
A
C
T
T
C
G
A
A
G
T
C
C
G
T
A
A
G
T
C
A
G
T
C
A
C
G
T
A
C
T
G
A
T
G
C
C
G
A
T
  

|  |  |
| --- | --- |
| p-value: | 1e-2301 |
| log p-value: | -5.299e+03 |
| Information Content per bp: | 1.750 |
| Number of Target Sequences with motif | 11192.0 |
| Percentage of Target Sequences with motif | 53.11% |
| Number of Background Sequences with motif | 2544.8 |
| Percentage of Background Sequences with motif | 20.85% |
| Average Position of motif in Targets | 270.9 +/- 222.3bp |
| Average Position of motif in Background | 294.9 +/- 263.8bp |
| Strand Bias (log2 ratio + to - strand density) | 0.1 |
| Multiplicity (# of sites on avg that occur together) | 1.36 |
| Motif File: | file (matrix) reverse opposite |

### Similar de novo motifs found

|  |  |  |  |  |  |  |  |
| --- | --- | --- | --- | --- | --- | --- | --- |
| Rank | Match Score | Redundant Motif | P-value | log P-value | % of Targets | % of Background | Motif file |
| 1 | 0.994 | C G T A T C G A G C T A T C G A A G T C C G T A A T G C G T A C A C G T A C T G A T G C C A G T | 1e-2289 | -5272.545078 | 56.12% | 23.21% | motif file (matrix) |
| 2 | 0.968 | G A C T T C A G G T A C C G T A A G T C A G T C A C G T A C T G | 1e-2227 | -5129.404847 | 59.87% | 26.62% | motif file (matrix) |
| 3 | 0.827 | A G C T A G T C C G T A A G T C A G T C A C G T A C T G A C G T | 1e-413 | -952.795172 | 30.86% | 18.40% | motif file (matrix) |
| 4 | 0.719 | A G T C C G T A A C T G A C T G A C G T A C T G A G T C C G T A A C T G A C T G A C G T A C T G | 1e-290 | -669.792836 | 15.22% | 7.71% | motif file (matrix) |
| 5 | 0.869 | A G C T A C G T A G T C C G T A A C T G A C T G A C G T A C T G A G C T T C A G | 1e-177 | -408.663118 | 9.60% | 4.87% | motif file (matrix) |
| 6 | 0.797 | A G T C C G T A C T A G C T A G A C G T T C A G C G T A C T A G A C G T A C T G | 1e-175 | -404.022776 | 18.34% | 11.65% | motif file (matrix) |
| 7 | 0.687 | C T A G C G A T T G A C A G T C C G T A C A G T G T C A A C G T C A T G A T C G | 1e-169 | -390.960434 | 37.22% | 28.36% | motif file (matrix) |
| 8 | 0.666 | T A G C G T A C C T G A T A C G G T C A G A C T A C T G A C T G | 1e-123 | -284.107240 | 20.01% | 14.05% | motif file (matrix) |
| 9 | 0.644 | G T C A C G T A T C A G A C T G A C G T C G T A A C T G C T G A | 1e-100 | -231.323764 | 36.85% | 29.99% | motif file (matrix) |
| 10 | 0.740 | C T A G A G T C A G T C C G T A A G T C A G T C A C G T C T A G A T C G A G T C | 1e-68 | -158.823920 | 3.34% | 1.60% | motif file (matrix) |
| 11 | 0.646 | A G T C A G T C G C T A C A T G A C T G C G A T C T A G A C T G A G T C A C G T | 1e-44 | -102.939282 | 3.53% | 2.03% | motif file (matrix) |
| 12 | 0.645 | A C T G G A T C G C T A A T G C A G T C C G A T A C T G C G T A G T C A C G T A A G T C G C A T | 1e-31 | -73.257882 | 0.32% | 0.06% | motif file (matrix) |
| 13 | 0.635 | A G T C A C T G A G T C C G T A C G T A A G T C C G A T A C T G A T C G C T A G | 1e-20 | -47.556577 | 0.19% | 0.03% | motif file (matrix) |
